# Denervation-Induced Activation of the Ubiquitin-Proteasome System Reduces Skeletal Muscle Quantity Not Quality

**DOI:** 10.1371/journal.pone.0160839

**Published:** 2016-08-11

**Authors:** Cory W. Baumann, Haiming M. Liu, LaDora V. Thompson

**Affiliations:** Department of Physical Medicine and Rehabilitation, University of Minnesota Medical School, Minneapolis, Minnesota, United States of America; University of Louisville School of Medicine, UNITED STATES

## Abstract

It is well known that the ubiquitin-proteasome system is activated in response to skeletal muscle wasting and functions to degrade contractile proteins. The loss of these proteins inevitably reduces skeletal muscle size (i.e., quantity). However, it is currently unknown whether activation of this pathway also affects function by impairing the muscle’s intrinsic ability to produce force (i.e., quality). Therefore, the purpose of this study was twofold, (1) document how the ubiquitin-proteasome system responds to denervation and (2) identify the physiological consequences of these changes. To induce soleus muscle atrophy, C57BL6 mice underwent tibial nerve transection of the left hindlimb for 7 or 14 days (n = 6–8 per group). At these time points, content of several proteins within the ubiquitin-proteasome system were determined via Western blot, while *ex vivo* whole muscle contractility was specifically analyzed at day 14. Denervation temporarily increased several key proteins within the ubiquitin-proteasome system, including the E3 ligase MuRF1 and the proteasome subunits 19S, α7 and β5. These changes were accompanied by reductions in absolute peak force and power, which were offset when expressed relative to physiological cross-sectional area. Contrary to peak force, absolute and relative forces at submaximal stimulation frequencies were significantly greater following 14 days of denervation. Taken together, these data represent two keys findings. First, activation of the ubiquitin-proteasome system is associated with reductions in skeletal muscle quantity rather than quality. Second, shortly after denervation, it appears the muscle remodels to compensate for the loss of neural activity via changes in Ca^2+^ handling.

## Introduction

Skeletal muscle atrophy can occur from a variety of stressors, which include inflammation, mechanical unloading, metabolic stress and neural inactivity. These stressors are often a result of clinical conditions or chronic diseases such as limb immobilization, bed rest, cachexia, neurodegeneration and aging [1–4]. Under these situations, there is an imbalance between anabolic and catabolic processes with protein breakdown (i.e., proteolysis) exceeding protein synthesis [5, 6]. Consequently, muscle mass is reduced due to the net loss of myofibrillar and soluble proteins, organelles and cytoplasm, all of which alter muscle function either metabolically, structurally or physiologically. The increased rate of proteolysis is mediated through the autophagy-lysosome and ubiquitin-proteasome systems [4, 7, 8], with the latter reported to be the primary degradation pathway in skeletal muscle [9, 10].

The ubiquitin-proteasome system is a complex pathway in skeletal muscle that consists of several components [1, 3, 11, 12]. Briefly, proteins destined for degradation are conjugated to multiple molecules of ubiquitin by an ubiquitin ligase enzyme (E3). One of the main E3 ligases in skeletal muscle is the muscle RING finger 1 (MuRF1) due to the fact many of its substrates are sarcomeric proteins. Thus far, MuRF1 has been observed to ubiquitinate/interact with troponin I [13], actin [14], myosin binding protein C [15], myosin light chains 1 and 2 [15], myosin heavy chain (MHC) [16, 17] and titin [18, 19]. These polyubiquitin-conjugated proteins are then recognized and subsequently degraded into peptides by the 26S proteasome. To note, the 26S proteasome is composed of a cylindrical catalytic core particle (20S) capped at one or both ends with 19S regulatory complexes (also known as PA700). These 20S particles consist of four rings that contain seven subunits each. The two outer rings are composed of α-subunits (α1 to α7) and the two inner rings of β-subunits (β1 to β7). Importantly, three of these β-subunits perform distinct proteolytic activities. Specifically, activity of β1 (PSMB6), β2 (PSMB7) and β5 (PSMB5) are classified as caspase-like, trypsin-like and chymotrypsin-like for their cleavage of acidic, basic and hydrophobic amino acids, respectively.

It is well established that several components within the ubiquitin-proteasome system increase in response to skeletal muscle wasting [20–23], and that these changes are responsible for degrading the myofibrillar proteins MHC and actin [9, 24]. Loss of these contractile proteins inevitably reduces skeletal muscle size (i.e., quantity). Consequently, a smaller muscle produces less force. For instance, it has been reported that absolute force and power were both reduced in atrophied skeletal muscle following extended periods of denervation (e.g., ≥one month) [25, 26]. Interestingly, contractile deficits were also observed when expressed relative to muscle size, indicating the muscle’s intrinsic ability to contract (i.e., quality) was impaired at these later time points [25, 26]. Although it is generally accepted loss of muscle quantity following denervation is due to the ubiquitin-proteasome system, it is currently unknown whether muscle quality is affected soon after an episode of increased proteolysis mediated by this pathway.

Therefore, the goals of this study were twofold, (1) document how the ubiquitin-proteasome system responds to denervation and (2) identify the physiological consequences of these changes. To accomplish this, we first determined when content of the ubiquitin-proteasome system returned back to resting levels (i.e., shortly following activation) in denervated skeletal muscle. At this point, we then performed *ex vivo* physiology to assess whole muscle contractility. Using this design, we were able to document how muscle function was affected soon after protein content of the ubiquitin-proteasome system was upregulated. We hypothesized that activation of the ubiquitin-proteasome system would be associated with reductions in skeletal muscle quantity rather than quality. If correct, these findings would demonstrate the ubiquitin-proteasome system is a fine-tuned proteolytic pathway that alters muscle size without significantly influencing its intrinsic ability to generate force.

## Methods

### Animals

Adult male C57BL6 mice (6.50±0.24 months old) were used in this study. Mice were housed in groups of no more than 4 animals per cage, supplied with food and water *ad libitum*, and maintained in a room at 20–23°C with a 12-h photoperiod. During the final procedure (i.e., ***Ex vivo* muscle preparation and contractility measurements**), mice were anesthetized by an intraperitoneal injection of ketamine/xylazine (100 mg/kg ketamine, 10 mg/kg xylazine), with supplemental doses given as required. Following the completion of this procedure, mice were euthanized by exsanguination while under anesthesia. All animal procedures were approved by the Institutional Animal Care and Use Committee at the University of Minnesota.

### Experimental design

Mice were randomly assigned to one of three groups: control, 7 day denervation or 14 day denervation. The control group consisted of mice that did not receive any surgery, while mice in the denervation groups underwent tibial nerve transection of the left hindlimb and were assessed in the following 7 or 14 days. Tibial nerve transection surgery results in denervation to the gastrocnemius, plantaris and soleus muscles, and has been reported to result in significant atrophy [27, 28]. However, only the soleus muscle of the left hindlimb was evaluated in this study. The soleus was specifically selected from the denervated muscles because it can be prepared for *ex vivo* physiology due to its accessibility and size. Biochemical analyses including Western blot, proteasome proteolytic activity and silver staining were assessed in control, 7 day and/or 14 day groups, while fiber cross-sectional area (CSA) and whole muscle physiology were examined in the control and 14 day groups. Post-denervation CSA and muscle physiology were only analyzed at day 14 because the goal of this study was to assess contractility once the ubiquitin-proteasome system returned back to baseline (i.e., following upregulation), which we determined via Western blot analysis and proteasome proteolytic activity (See Results).

### Experimental methodology

#### Tibial nerve transection

Tibial nerve transection was performed on the left hindlimb, similar to that previously described [27, 29]. Briefly, while under anesthesia (2.5% isoflurane), an incision of ~1 cm was made from the sciatic notch to the knee. Following this initial incision, another small incision was made through the hamstring muscles to allow access to the tibial nerve. The tibial nerve was then separated from the peroneal and sural nerve branches at the area of the popliteal fossa. Using an 8–0 sterile silk suture, knots were made around the distal and proximal ends of the tibial nerve, separated by ~5 mm. A ≥3 mm piece of nerve between the two knots was then removed and the proximal end of the tibial nerve was sutured to the biceps femoris to prevent re-innervation. After denervation, the incisions made in the muscle and skin were sutured together and glued with vet-bond. Following the surgery, mice were given 0.1 ml Buprenorphine (0.03 mg/ml) for analgesia and monitored until they were ambulatory. Mice were brought back to the laboratory 7 or 14 days post-surgery.

#### *Ex vivo* muscle preparation and contractility measurements

The soleus muscle from the left hindlimb of an anesthetized mouse was dissected free and studied using an *ex vivo* preparation, similar to that described previously [30–32]. Following excision, the muscle was mounted in an organ bath containing a Krebs-Ringer buffer (pH 7.3) with 115 mM NaCl, 5.9 mM KCl, 1.2 mM MgCl_2_, 1.2 mM NaH_2_PO_4_, 1.2 mM Na_2_SO_4_, 2.5 mM CaCl_2_, 25 mM NaHCO_3_ and 10 mM glucose, which was equilibrated with 95% O_2_-5% CO_2_ gas and maintained at 25°C via a circulating water system. The distal tendon was attached by a silk suture and secured to a fixed support, and the proximal tendon was attached to the lever arm of a servomotor system (300B; Aurora Scientific Inc., Aurora, ON, Canada). Optimal muscle length (L_o_) in the organ bath was set with a series of twitch stimulations (0.2 ms pulse at 30 V). *Ex vivo* muscle length was then measured from the proximal to the distal myotendinous junctions using a digital caliper. Three minutes after L_o_ was determined, the muscle performed a final twitch contraction followed by a force-frequency protocol that included five isometric contractions (900 ms train of 0.2 ms pulses at 10, 40, 80, 100 and 120 Hz), all separated with a 3 min rest period. For analysis, the final twitch was recorded as peak isometric twitch force (P_t_), whereas the highest recorded force during the force-frequency protocol was defined as peak isometric tetanic force (P_o_).

Force-frequency relationships were modeled with the following equation: *f*(*x*) = min + (max−min) / [1 + (*x*/ EC_50_)^*n*^], where *x* is the stimulation frequency, min and max are the smallest (i.e., twitch) and largest (i.e., peak tetanic) respective estimated forces, EC_50_ is the stimulation frequency at which half the amplitude of force (max − min) is reached and *n*, the Hill coefficient, characterizes the slope of the curve at its midpoint.

After P_o_ was determined, the load clamp technique was used to determine velocity of contraction as previously described [33, 34]. In brief, the muscle length was set at L_o_ and activated at various percentages of P_o_ (10, 20, 30, 40, 60, 70, 80 and 90%) using the stimulation frequency that corresponded to P_o_ for a 500 ms train. Once the muscle could overcome the set load (clamped at 10–90% of P_o_), the muscle concentrically contracted and the distance the servomotor level arm moved was recorded. The contraction velocity for each % load of P_o_ was then computed and converted from millimeters per second (mm/s) to a normalized measurement of fiber lengths per second (fl/s) using a ratio of intact fiber length to muscle length of 0.69 [35]. Using a custom MATLAB (Natick, MA) program, a force-velocity curve was generated using the hyperbolic Hill equation and the maximum unloaded velocity (V_max_) was determined by extrapolation to the zero load of the force-velocity relationship. From the force and velocity data, a force-power curve was constructed in MATLAB (by fitting the data to a 5th degree polynomial curve) and peak power (P_max_) and the percentage of P_o_ at which P_max_ occurred (% P_o_ at P_max_) were calculated.

Following the force-velocity protocol, the soleus muscle was removed, trimmed, weighed and stored at −80°C for later analysis. Physiological cross-sectional area (PCSA) was computed using the average density of skeletal muscle: PCSA (cm^2^) = muscle mass (g)/[L_o_ (cm)*1.06 (g/cm^3^)] [36]. To attain relative force and power values, all absolute values were normalized to PCSA (cm^2^).

#### Histology

In mice that were not used for the *ex vivo* physiology experiments, the soleus muscles were excised, embedded in a tissue freezing medium and immediately frozen in 2-methylbutane cooled in liquid nitrogen. Transverse, serial sections at 10 μm were cut through the mid-belly of the soleus muscle using a cryostat (Leica CM3050S, Nussloch, Germany). Sections were then processed for hematoxylin and eosin staining, dehydrated, mounted and visualized at 10X with a Nikon Eclipse E400 microscope (Nikon, Tokyo, Japan) as previously described [33]. The CSA of approximately 300 fibers per muscle was calculated using ImageJ analysis software (National Institutes of Health, Bethesda, MD, USA).

#### Western blotting

Left soleus muscles were homogenized in an ice-cold RIPA lysis and extraction buffer (Thermo Scientific, Rockford, IL) supplemented with a protease inhibitor cocktail (Thermo Scientific). Total protein content was quantified with a bicinchoninic acid (BCA) assay (Thermo Scientific) using bovine serum albumin (BSA) as a standard. A portion of the muscle homogenate was then diluted in a loading buffer and heated for 4 min. Equal amounts of protein (25 μg) were loaded onto a 10% SDS polyacrylamide gel and separated according to molecular weight (100 V for 120 min). The proteins were then transferred to a PDVF membrane using a semi-dry transfer system at 15 V for 30 min (Bio-Rad Laboratories, Hercules, CA). Membranes were allowed to dry overnight and then blocked in 5% nonfat dried milk (w/v) dissolved in tris-buffered saline with 0.1% Tween-20 (TBS-T) for 1 hour at room temperature. Following the block, the membranes were probed with an anti-MuRF1 (1:250; #32920, Santa Cruz), anti-ubiquitin (1:2000; #3933, Cell Signaling), anti-19S Rpt1/S7 subunit (referred to as 19S in the present study) (1:1000; #PW9400, Enzo Life Sciences), anti-α7 (1:1000; #PW8110, Enzo Life Sciences), anti-β5 (1:1000; #PA1-977, Thermo Scientific), anti-GAPDH (1:5000; #3683, Cell Signaling) or an anti-α-tubulin (1:2000, #2144, Cell Signaling) primary antibody diluted in 0.2% nonfat dried milk dissolved in TBS-T for 2 h at room temperature on an orbital shaker. Following incubation in the primary antibodies, membranes were washed with TBS-T (3×5 min) and then probed with the appropriate secondary antibody (goat anti-rabbit IgG, 1:10000; Santa Cruz or goat anti-mouse IgG, 1:10000; Thermo Scientific) in 5% nonfat dried milk dissolved in TBS-T for 1 h at room temperature with shaking and washed as previously stated. Membranes were then treated with an enhanced chemiluminescent solution (Thermo Scientific) prior to detection using a BioRad ChemiDoc XRS imaging station (Bio-Rad Laboratories) and analyzed by densitometry using QuantityOne software (Bio-Rad Laboratories).

To note, GAPDH and α-tubulin were initially selected to serve as loading controls; however, were not used because they significantly changed as a result of denervation (See Results). Thus, to ensure protein load was similar between samples, all membranes were stained with Ponceau S before blocking or Coomassie Blue after imaging.

#### Proteasome proteolytic activity

Because β5 performs chymotrypsin-like activity we specifically determined its proteolytic activity by measuring degradation of fluorogenic peptide substrates as previously described [37]. In brief, 10 μg of the muscle homogenate (prepared as described in **Western blotting** with the exception of the protease inhibitor cocktail) was incubated either with or without the proteasome inhibitor MG132 (0.2 mM) (Peptides International, Louisville, KY) in 100 μl of a 50 mM Tris buffer (pH 7.8) for 30 min at 37°C. After incubation, 100 μL of a fluorogenic peptide mixture containing 75 μM of LLVY-AMC (Proteasome Substrate III, Fluorogenic, Millipore, Billerica, MA) dissolved in a 40 mM Tris (pH 7.5) buffer supplemented with 20 mM KCL, 10 mM MgCl_2_, and 0.5 mM ATP was added to each sample. To note, the fluorogenic peptide LLVY-AMC is used to model chymotrypsin-like activity. Fluorescence was measured at 37°C in a Synergy^™^ HTX Multi-Mode Microplate Reader (BioTek, Winooski, VT) at a wavelength of 360/40 nm (excitation), 460/40 nm (emission) and a gain of 70 for 2 h at 5 min intervals. Chymotrypsin-like activity was determined by comparing peptide fluorescence from samples with fluorescence of a standard curve of 7-amino-4-methylcoumarin (AMC, Sigma-Aldrich, St. Louis, MO). The difference between assays with or without MG132 represented the proteasome-specific chymotrypsin-like activity and was used for analysis.

#### Determination of contractile protein content (Silver staining)

Protein content of myosin heavy chain (MHC) and actin were estimated using a gel silver staining kit (Bio-Rad) as previously described [38]. Briefly, equal amounts of protein (2 μg) were loaded onto an 8% SDS polyacrylamide gel and separated according to molecular weight (100 V for 100 min). Following electrophoresis, gels were fixed for 20 min and decanted with deionized distilled water for 20 min (2×10 min). Gels were then silver stained for 16–21 min, transferred into a 5% acetic acid solution for 15 min to stop the reaction and rinsed with deionized distilled water for 20 min (2×10 min). The stained gels were imaged using a BioRad GS-800 imager (Bio-Rad) and analyzed by densitometry using QuantityOne software (Bio-Rad). Specifically, content within each gel lane was determined, which provided an estimation of the relative amount of MHC and actin per sample.

### Statistics analysis

An independent t-test or one-way ANOVA was used to determine differences between groups when two (control vs. 14 day) or three (control vs. 7 day vs. 14 day) independent variables were selected, respectively. To assess changes between groups across force-frequency, force-velocity and force-power curves, all data (group × Hz or % P_o_) were entered into a two-way repeated measures ANOVA. A Fisher’s least significant difference (LSD) *post hoc* test was performed in the event of a significant ANOVA. An α-level of ≤0.05 was used for all analyses. Values are presented in mean±SEM. All statistical testing was performed using SigmaPlot version 11.0 (Systat Software, San Jose, CA).

## Results

### Activation of the ubiquitin-proteasome system

**Fig 1:**
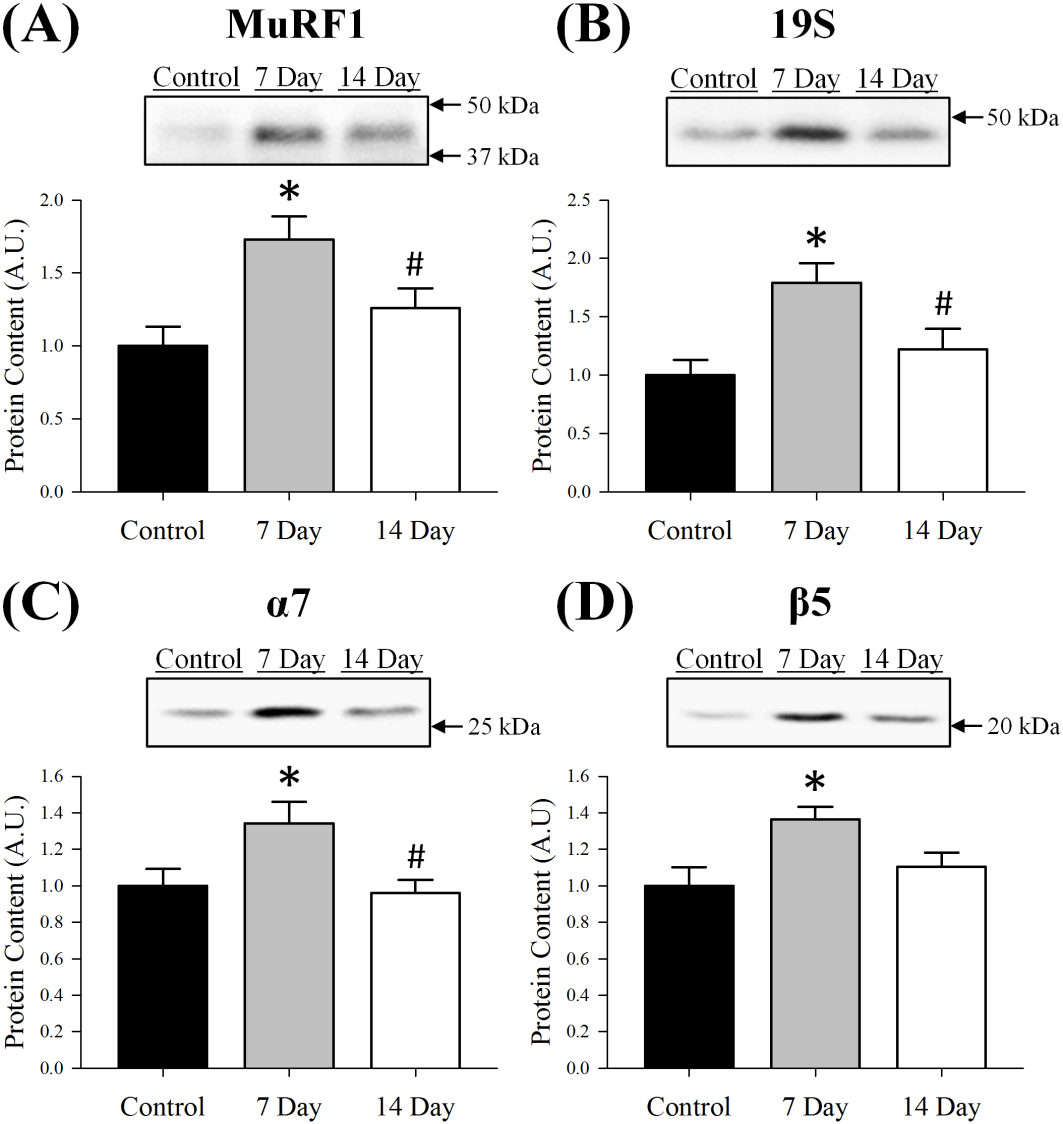
Protein content of (A) MuRF1, (B) 19S, (C) α7 and (D) β5 in control, 7 day or 14 day soleus muscle. Content of each protein was determined using Western blot analysis, normalized to the control muscle and set at 1.0. Pictured above the graphs are representative Western blots for each protein with arrows indicating molecular weight standards. All lanes were loaded with an equal amount of total protein (25 μg). Control was innervated muscle, 7 day was denervated for 7 days and 14 day was denervated for 14 days. Sample size per group, n = 5–7. Values are mean±SEM. *Significantly different from control (p≤0.05). ^#^Significantly different from 7 day (p≤0.05). A.U., arbitrary units.

**Fig 2:**
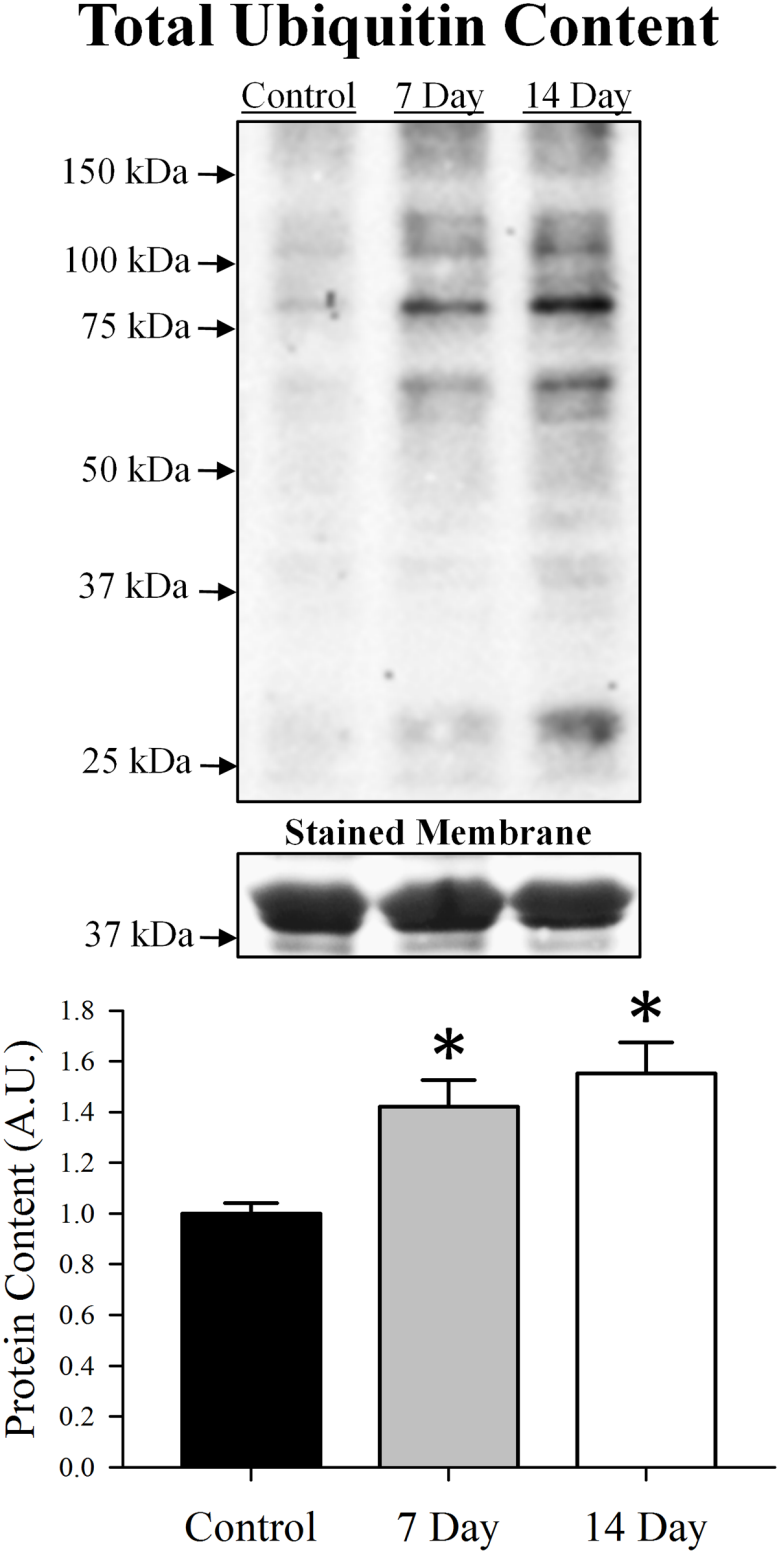
Total ubiquitin content in control, 7 day or 14 day soleus muscle. Content of each protein was determined using Western blot analysis, normalized to the control muscle and set at 1.0. Pictured above the graph is a representative Western blot with arrows indicating molecular weight standards. The stained membrane is a representative section (above ~37 kDa) of a Coomassie Blue stained membrane. All lanes were loaded with an equal amount of total protein (25 μg). Control was innervated muscle, 7 day was denervated for 7 days and 14 day was denervated for 14 days. Sample size per group, n = 5–7. Values are mean±SEM. *Significantly different from control (p≤0.05). A.U., arbitrary units.

To assess how the ubiquitin-proteasome system responded to denervation, we used Western blot analysis to determine content of several key proteins implicated in this pathway (Figs 1 and 2). The first protein assessed was the E3 ligase MuRF1 because it can interact with several key sarcomeric proteins. As previously reported, MuRF1 content was relatively low under basal conditions, but increased as a result of denervation [15]. By 7 days post-denervation, MuRF1 content was elevated 73% (p = 0.003; Fig 1A) and was accompanied by a 42% (p = 0.008; Fig 2) increase in total ubiquitin content. At day 14, MuRF1 content returned to baseline (p = 0.19) while total ubiquitin content remained elevated (55%, p = 0.001). At both 7 and 14 days post-denervation, most of the ubiquitinated proteins were observed just above 25 kDa and between 75 to150 kDa.

Next, we assessed different components of the 26S proteasome by measuring a subunit of the 19s regulatory complex and two subunits of the 20S catalytic core, α7 and β5 (Fig 1B–1D). The changes in proteasome content were similar to that of MuRF1 and ubiquitin at day 7. Specifically, 19S, α7 and β5 content increased 79% (p = 0.004), 34% (p = 0.022) and 36% (p = 0.012), respectively. By day 14, 19S, α7 and β5 content decreased and did not differ from control muscle (p≥0.32). As expected, chymotrypsin-like activity of the proteasome was back to baseline 14 days post-denervation (control: 17.82±1.91 vs. 14 day: 23.27±3.13 pmol·mg^-1^·min^-1^, p = 0.19), comparable to that of β5 content.

### Skeletal muscle atrophy and contractile function

**Fig 3:**
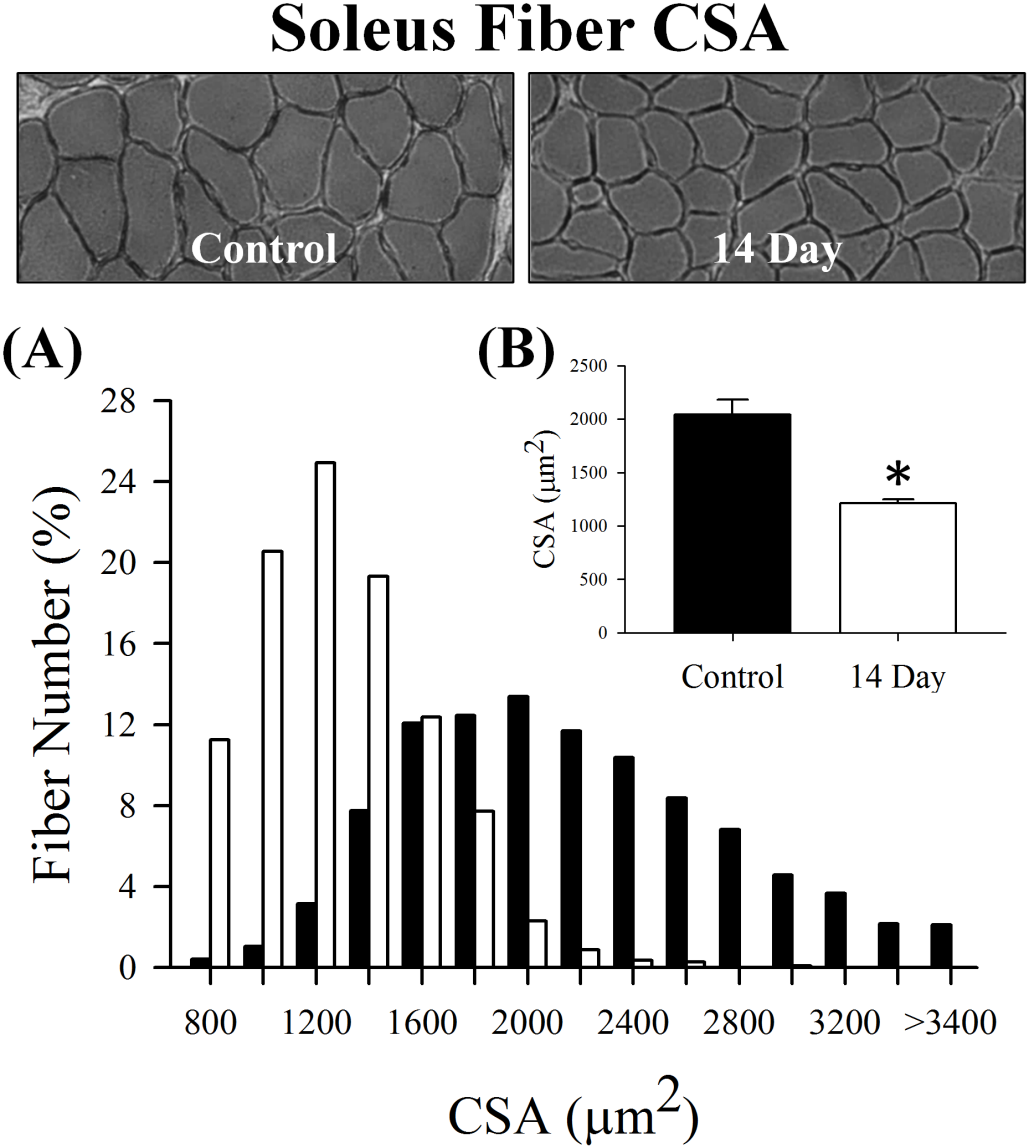
Fiber (A) distribution and (B) mean cross-sectional area (CSA) in control and 14 day soleus muscle. Pictured above the graphs are representative hematoxylin and eosin stains. Control was innervated muscle and 14 day was denervated for 14 days. Sample size per group, n = 6–7. Values are mean±SEM. *Significantly different from control (p≤0.05).

**Fig 4:**
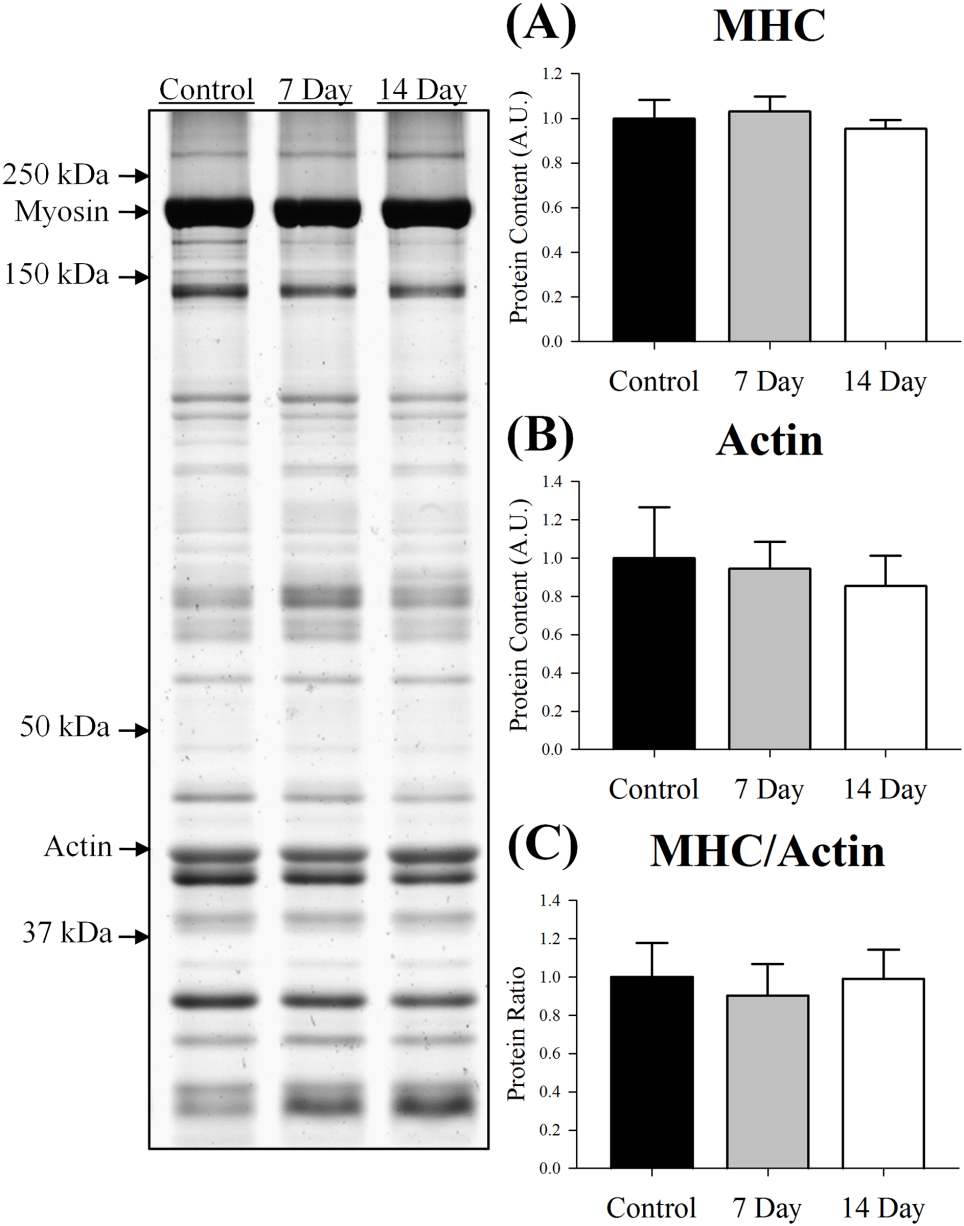
Protein content of (A) myosin heavy chain: MHC, (B) actin and (C) MHC/actin in control, 7 day or 14 day soleus muscle. Content of each protein was determined using gel silver staining, normalized to the control muscle and set at 1.0. Pictured to the left of the graphs is a representative silver stained gel with arrows indicating molecular weight standards. All lanes were loaded with an equal amount of total protein (2 μg). Therefore, these results depict that although denervation reduced fiber size (Fig 3), the relative concentration of MHC, actin and MHC/actin remained similar between the control and denervated muscle. Control was innervated muscle, 7 day was denervated for 7 days and 14 day was denervated for 14 days. Sample size per group, n = 5–7. Values are mean±SEM. A.U., arbitrary units.

Soleus wet weight and fiber CSA were reduced by 28% and 41% by day 14 (p≤0.001; Fig 3), respectively. Both of which indicate myofibrillar protein content declined following denervation. Therefore, we sought to determine if these reductions were due to a preferential loss of MHC or actin by assessing silver stained gels that were loaded with equal amounts of protein (2 μg). Despite the loss of muscle wet weight and fiber CSA (Fig 3), the relative concentration of MHC, actin and MHC/actin remained similar between the control and denervated muscle (p≥0.72; Fig 4). This observation suggests that MHC and actin content were lost at comparable rates when assessed at 7 and 14 days post-denervation.

**Fig 5:**
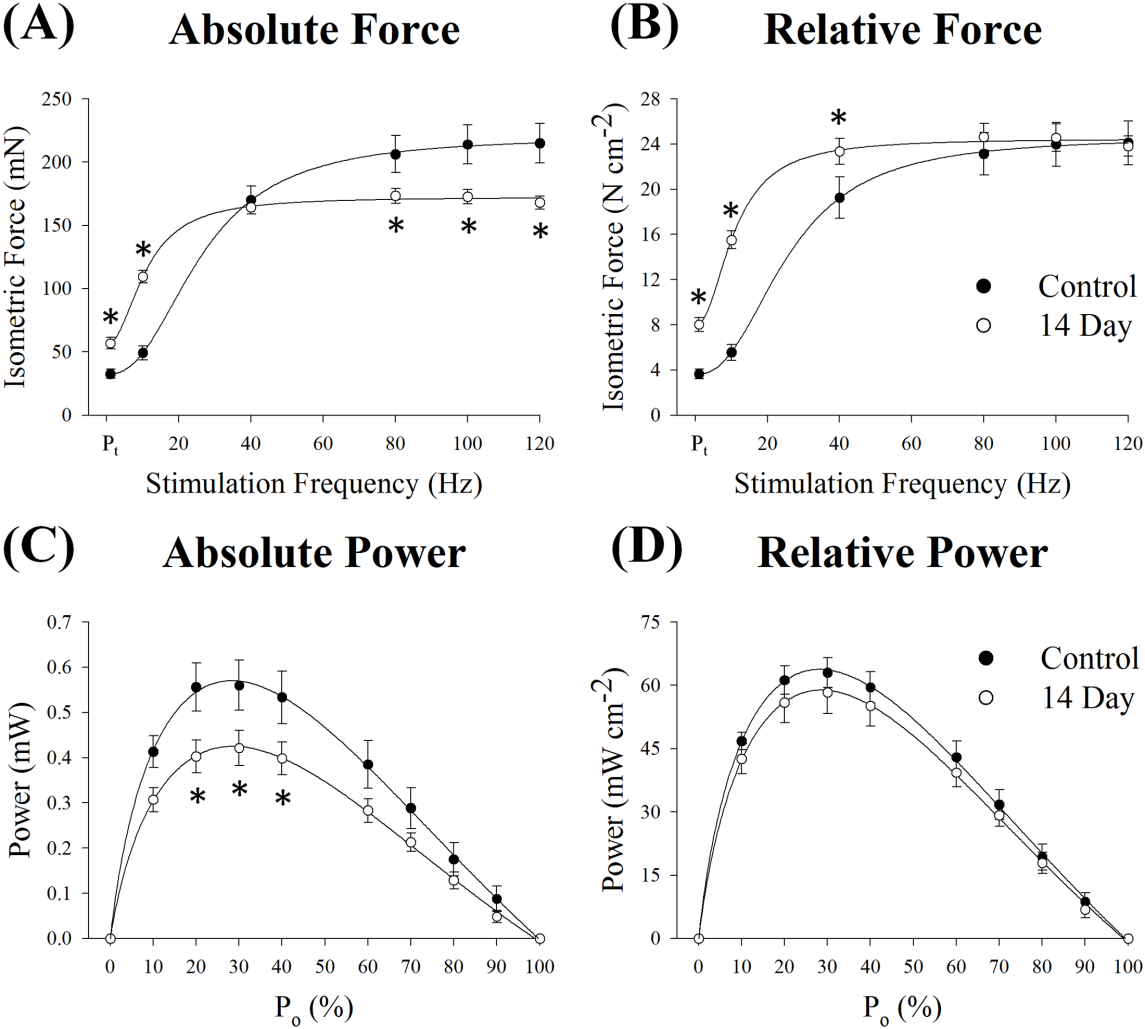
*Ex vivo* (A) absolute force, (B) relative force, (C) absolute power and (D) relative power in control and 14 day soleus muscle. Control was innervated muscle and 14 day was denervated for 14 days. Sample size per group, n = 6–8. Values are mean±SEM. *Significantly different from control (p≤0.05). P_t_, peak isometric twitch force.

Due to the loss of muscle wet weight and fiber CSA observed at day 14 (Fig 4), it was apparent contractile function would also be reduced, particularly when expressed in absolute terms (i.e., not relative to PCSA). As hypothesized, absolute P_o_ decreased 19% (p = 0.021; Fig 5A and Table 1) while relative P_o_ remained unchanged (p = 0.80; Fig 5B and Table 1).

**Table 1:** Soleus muscle characteristics and *ex vivo* contractile parameters.

	Control	Denervated
**Muscle weight (mg)**	10.76±0.51	7.72±0.25*
**Histological CSA (μm**^**2**^**)**	2040.88±139.86	1210.32±39.66*
**Muscle length (mm)**	10.82±0.33	10.60±0.44
**PCSA (mm**^**2**^**)**	0.92±0.08	0.71±0.02*
**Absolute P**_**t**_ **(mN)**	32.59±3.64	56.73±4.56*
**Absolute P**_**o**_ **(mN)**	215.28±15.57	173.68±5.83*
**Relative P**_**t**_ **(N cm**^**-2**^**)**	3.65±0.41	8.01±0.61*
**Relative P**_**o**_ **(N cm**^**-2**^**)**	24.10±1.94	24.69±1.22
**TPT (ms)**	36.43±1.88	56.71±1.60*
**RT**_**1/2**_ **(ms)**	40.29±1.25	66.14±5.20*
**EC**_**50**_ **(Hz)**	26.20±1.32	11.04±0.57*
**Hill coefficient**	2.48±0.12	1.89±0.06*
**V**_**max**_ **(fl/s)**	4.40±0.26	4.34±0.20
**Absolute P**_**max**_ **(mW)**	0.57±0.05	0.43±0.04*
**Relative P**_**max**_ **(mW cm**^**-2**^**)**	63.93±3.26	58.96±5.03
**% P**_**o**_ **at P**_**max**_ **(%)**	26.33±1.05	26.71±0.71

Values are means±SEM. Sample size per group, n = 19–20 for muscle weight. For all other variables, n = 6–8. The control group consisted of soleus muscle that was innervated, while the denervated group included soleus muscle that was denervated for 14 days. CSA, cross-sectional area; PCSA, physiological cross-sectional area; P_t_, peak isometric twitch force; P_o_, peak isometric tetanic force; TPT, time to P_t_; RT_1/2_, one-half P_t_ relaxation time; EC_50_, frequency at which 50% P_o_ was developed; Hill coefficient, slope coefficient of the force-frequency curve at its midpoint; V_max_, maximum unloaded velocity; P_max_, peak power.

* Significantly different from control (p≤0.05).

Unexpectedly, P_t_ did not mimic that of P_o_, as absolute and relative P_t_ both increased in the denervated muscle (74%, p = 0.001 and 120%, p≤0.001; Fig 5A, 5B and Table 1). To further assess these changes, time to P_t_ (TPT) and one-half relaxation time (RT_1/2_) were also analyzed (Table 1). As with P_t_, both increased at day 14 (~60% p≤0.001), which indicates a slowing of the contraction after denervation.

Additionally, there was a leftward shift in the force-frequency curves following denervation, which was accompanied by reductions in EC_50_ and the Hill coefficient (p≤0.001; Fig 5A, 5B and Table 1). Upon further analysis of the force-frequency curves, it was also apparent denervation affected absolute and relative force at several other stimulation frequencies, besides those that elicited P_t_ and P_o_. Absolute force at 10 Hz increased (p<0.001) while force at 80, 100 and 120 Hz (p≤0.016) decreased 14 days post-denervation. Moreover, relative force increased at 10 and 40 Hz (p≤0.037) but did not differ from control muscle at higher frequencies (p≥0.42).

Muscle power is the product of force and contraction velocity. Despite the changes observed in the force-frequency curves, denervation had no effect on V_max_ (p = 0.84, Table 1) or the force-velocity relationship (p = 0.99, S1 Fig). Peak power (P_max_) occurred at ~26% of P_o_ and was not different 14 days post-denervation (p = 0.77; Fig 5C, 5D and Table 1). However, absolute P_max_ decreased 25% (p = 0.050), comparable to the 19% reduction in absolute P_o_ previously stated. A similar reduction was also observed at 20, 30 and 40% of absolute P_o_ 14 days post-denervation (p≤0.029). As with relative P_o_, denervation did not alter relative P_max_ (p = 0.44) or any other relative % P_o_ on the force-power curve (p = 0.98).

### GAPDH and α-tubulin protein content

**Fig 6:**
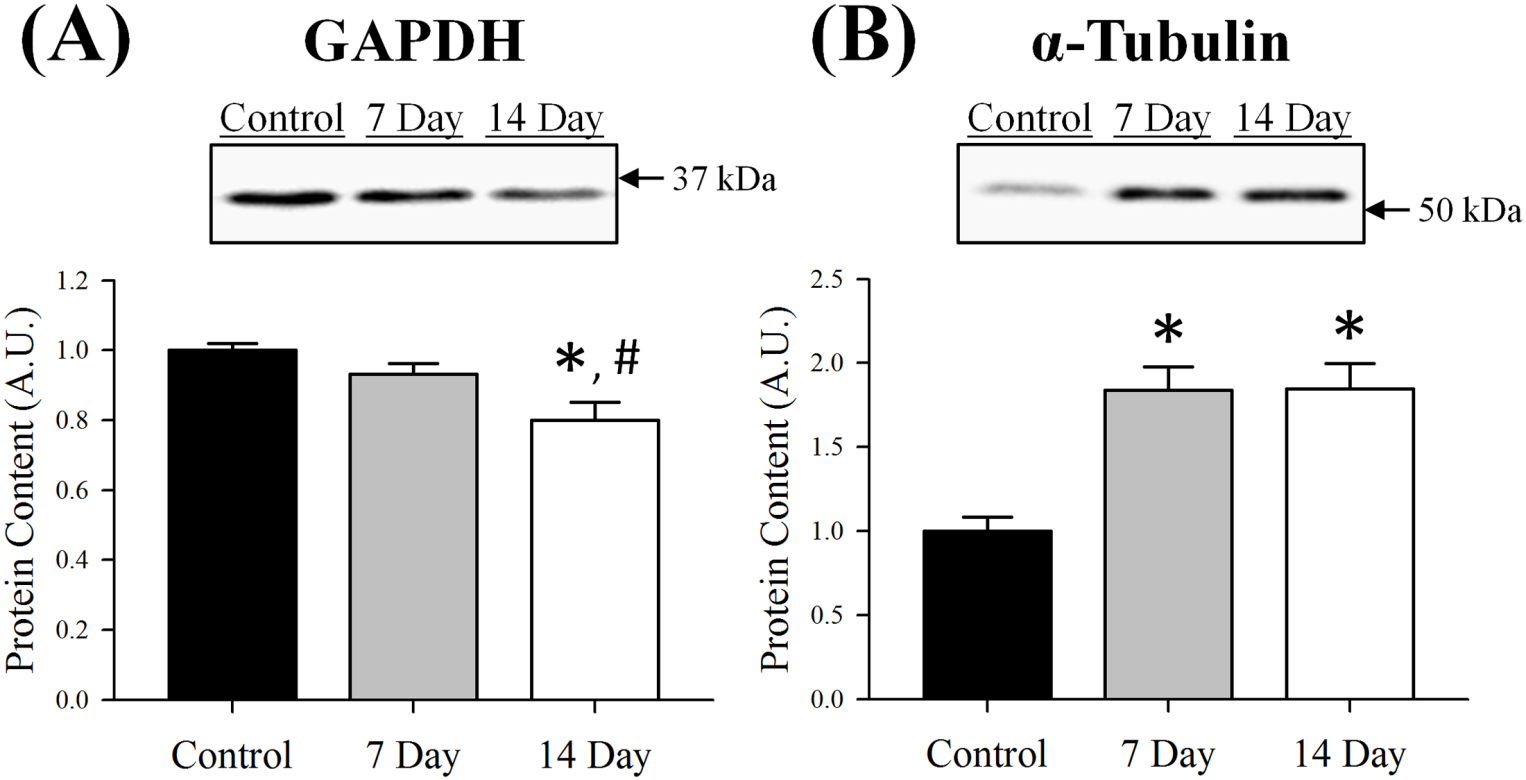
Protein content of (A) GAPDH and (B) α-tubulin in control, 7 day or 14 day soleus muscle. Content of each protein was determined using Western blot analysis, normalized to the control muscle and set at 1.0. Pictured above the graphs are representative Western blots for each protein with arrows indicating molecular weight standards. All lanes were loaded with an equal amount of total protein (25 μg). Control was innervated muscle, 7 day was denervated for 7 days and 14 day was denervated for 14 days. Sample size per group, n = 5–7. Values are mean±SEM. *Significantly different from control (p≤0.05). ^#^Significantly different from 7 day (p≤0.05). A.U., arbitrary units.

Content of GAPDH did not differ from control muscle 7 days following denervation (p≥0.14); however, decreased 20% by day 14 (p = 0.001, Fig 6A). Contrary to GAPDH, content of α-tubulin dramatically increased 7 days post-denervation and remained elevated through day 14 (~84%, p≤0.001; Fig 6B). It is worth noting that GAPDH and α-tubulin are common housekeeping proteins used by many as loading controls. However, as we and others report [39–41], these proteins are significantly influenced by denervation and therefore, if set as loading controls, caution should be used.

## Discussion

Skeletal muscle atrophy is primarily due to the loss of myofibrillar proteins and is a hallmark outcome of many clinical conditions and chronic diseases. The increase in proteolysis is largely attributed to the ubiquitin-proteasome system, the primary pathway responsible for degrading the contractile apparatus. Despite the correlative relationship between the ubiquitin-proteasome system and skeletal muscle quantity, it is unclear how muscle quality is affected soon after an episode of increased proteolysis mediated by this pathway. Therefore, we denervated mouse soleus muscle for up to 14 days and assessed content of the ubiquitin-proteasome system, in addition to *ex vivo* whole muscle contractility. Here, we report content of several key proteins within the ubiquitin-proteasome system temporarily increased following denervation and as a result, reduced skeletal muscle size. These changes were accompanied by reductions in absolute P_o_ and P_max_, which were offset when expressed relative to PCSA. However, contrary to P_o_, absolute and relative P_t_ increased as a result of denervation. Taken together, these data represent two key findings. First, activation of the ubiquitin-proteasome system is associated with reductions in skeletal muscle quantity rather than quality. Second, despite the loss of contractile proteins, short-term denervation increased force at submaximal stimulation frequencies, which suggests Ca^2+^ handling was enhanced possibly to compensate for the loss of neural activity.

Denervation-induced muscle atrophy models are known to increase several components of the ubiquitin-proteasome system, including E3 ligases, content of ubiquitinated proteins and several subunits of the 26S proteasome. Upregulation of the ubiquitin-proteasome system has been reported to increase as early as 2–3 days post-denervation [20–22] and remain elevated up to 14 days [22, 23]. Due to the complexity of this pathway and the various E3 ligases and proteasome subunits present in skeletal muscle, many of these studies assessed specific parts of the ubiquitin-proteasome system rather than it as a whole. However, it is apparent that these components (i.e., E3 ligases and proteasome subunits) are regulated in a coordinated response to mediate skeletal muscle proteolysis [21] and are controlled by similar transcription factors [8, 20]. Our analysis included key proteins from several different components of the ubiquitin-proteasome system: the E3 ligase MuRF1, total ubiquitin content and three subunits from different rings of the 26S proteasome (i.e., 19S, α7 and β5). As reported by others [20–23], protein content (or mRNA expression) of the ubiquitin-proteasome system significantly increased following denervation. Specifically, MuRF1, ubiquitin, 19S, α7 and β5 content were elevated 7 days post-denervation and returned to control levels by day 14, with the exception of ubiquitin. Other E3 ligases like NEDD4, which are not associated with sarcomeric proteins, have been found to be more persistent in denervated muscle [22, 28, 39] and may contribute to the presence of these ubiquitinated proteins beyond day 7, after MuRF1 and proteasome content have declined.

The temporary increase in MuRF1, 19S, α7 and β5 content was associated with reductions in soleus muscle wet weight and fiber CSA after 14 days of denervation. Loss of muscle size is thought to be primarily due to the proteolysis of MHC and actin by the ubiquitin-proteasome system [9]. Importantly, when loading equal amounts of protein (2 ug), the ratio of MHC to actin did not change following 7 or 14 days of denervation, illustrating MHC and actin were degraded at similar rates. Comparable results have previously been reported in mouse gastrocnemius muscle after 10 days of denervation [15]. In support of our MHC to actin ratio, absolute P_o_ and P_max_ were reduced while relative P_o_ and P_max_ were unaffected, which suggests the muscle’s intrinsic ability to generate force was not influenced at day 14. Furthermore, denervation did not significantly alter V_max_ indicating the decrease in P_max_ was primarily due to P_o_, or more accurately, degradation of the myofibrillar proteins and thus loss of fiber CSA. These contractile observations (i.e., P_o_, P_max_ and V_max_) confirm that of Greising et al. [27], who used tibial nerve transection and assessed soleus muscle from ovariectomized female mice following 14 days of denervation.

However, at later time points, others have shown the muscle’s intrinsic ability to generate force inevitability declines [25, 26]. For instance, Agbulut et al. [25] demonstrated that after one month of denervation, mouse soleus muscle exhibits marked reductions in V_max_ and relative P_o_ and P_max_ when compared to innervated control muscle. Taken together, the present findings and that of others [25, 26] suggest muscle quality is maintained soon after the ubiquitin-proteasome system has been activated, yet in the ensuing weeks muscle quality steadily declines. We propose that the reductions observed in muscle quality after extended periods of denervation (e.g., ≥one month) are due to factors other than the ubiquitin-proteasome system. Although these factors were not determined in the present study, others have shown denervation eventually affects muscle quality by reducing the fraction of CSA occupied by the contractile proteins [42–44] and through decreasing the fibers’ sensitivity to Ca^2+^ [45].

In addition to these findings, the fact that both absolute and relative submaximal force (≤10 Hz and ≤40 Hz, respectively) increased following denervation indicates Ca^2+^ handling was enhanced. When specifically assessing the twitch contraction, we observed an overall improvement of 74% in absolute force and 120% in relative force, which was accompanied by a 60% increase in TPT and RT_1/2_. Although these changes (i.e., twitch parameters) have not been reported in mouse soleus muscle at this specific time point, they are consistent with that seen in rat soleus muscle following 7 days of denervation [46]. These results suggest Ca^2+^ kinetics, such as Ca^2+^ release, diffusion and binding, were influenced shortly after skeletal muscle denervation [47]. In support of this, in the aforementioned rat soleus muscle, sarcoplasmic reticulum (SR) Ca^2+^ release was markedly elevated after 7 days of denervation and correlated with the slowing of the twitch contraction. With more time to develop force and Ca^2+^ available to bind with troponin C, P_t_ will ultimately increase as we and other have observed [26, 46].

Notably, these changes occurred during a period of significant proteolysis, as seen by the loss of both muscle mass and CSA, which suggests the excitation-contraction (EC) coupling pathway may undergo remodeling following denervation [48]. Some important features within the EC coupling pathway of denervated rodent muscle that could influence Ca^2+^ handling, include acetylcholine receptor expression, density and sensitivity [49, 50], dihydropyridine receptor expression [51–53] and SR Ca^2+^ loading capacity [46, 47]. Interestingly, similar observations (i.e., upregulation of the acetylcholine and dihydropyridine receptors) have been reported in the days following eccentric contraction-induced injury when EC coupling failure is pervasive [30, 54, 55]. These changes may reflect a compensatory mechanism to counteract the loss in voltage-induced SR Ca^2+^ release that occurs after eccentric contractions (i.e., 0–5 days post-injury) or denervation in skeletal muscle. However, P_t_ has been shown to progressively decline following extended periods of denervation, despite TPT and RT_1/2_ remaining prolonged [25, 26]. We propose that the changes observed in Ca^2+^ handling are negated by the persistent loss of the myofibrillar apparatus and/or eventual reduction in the muscle’s quality.

In closing, we demonstrate that several key proteins of the ubiquitin-proteasome system including MuRF1, 19S, α7 and β5 were all upregulated in mouse soleus muscle after denervation. Content of these proteins increased in a coordinated fashion and were only temporally affected by denervation. These changes were associated with reductions in muscle size and maximal force, but not muscle quality, at least at 14 days post-denervation. In contrast, submaximal force was improved despite the loss of MHC and actin, which suggests Ca^2+^ handling was enhanced. Taken together, our results demonstrate the ubiquitin-proteasome system is a fine-tuned proteolytic pathway, which upon activation primarily impairs maximal force by reducing skeletal muscle size, rather than its intrinsic ability to generate force. In addition, we also suggest the EC coupling pathway undergoes remodeling in concert to the proteolysis that occurs shortly following denervation, possibly to compensate for the loss in voltage-induced SR Ca^2+^ release.

## Supporting Information

S1 Fig *Ex vivo* force-velocity relationship in control and 14 day soleus muscle. Control was innervated muscle and 14 day was denervated for 14 days. Sample size per group, n = 6–8. Values are mean±SEM.
(TIF)
