# Supplementary figures and images for: Denervation-Induced Activation of the Ubiquitin-Proteasome System Reduces Skeletal Muscle Quantity Not Quality
Source: PLoS One. 2016 Aug 11;11(8):e0160839. doi: 10.1371/journal.pone.0160839 (PMC4981385; doi:10.1371/journal.pone.0160839)

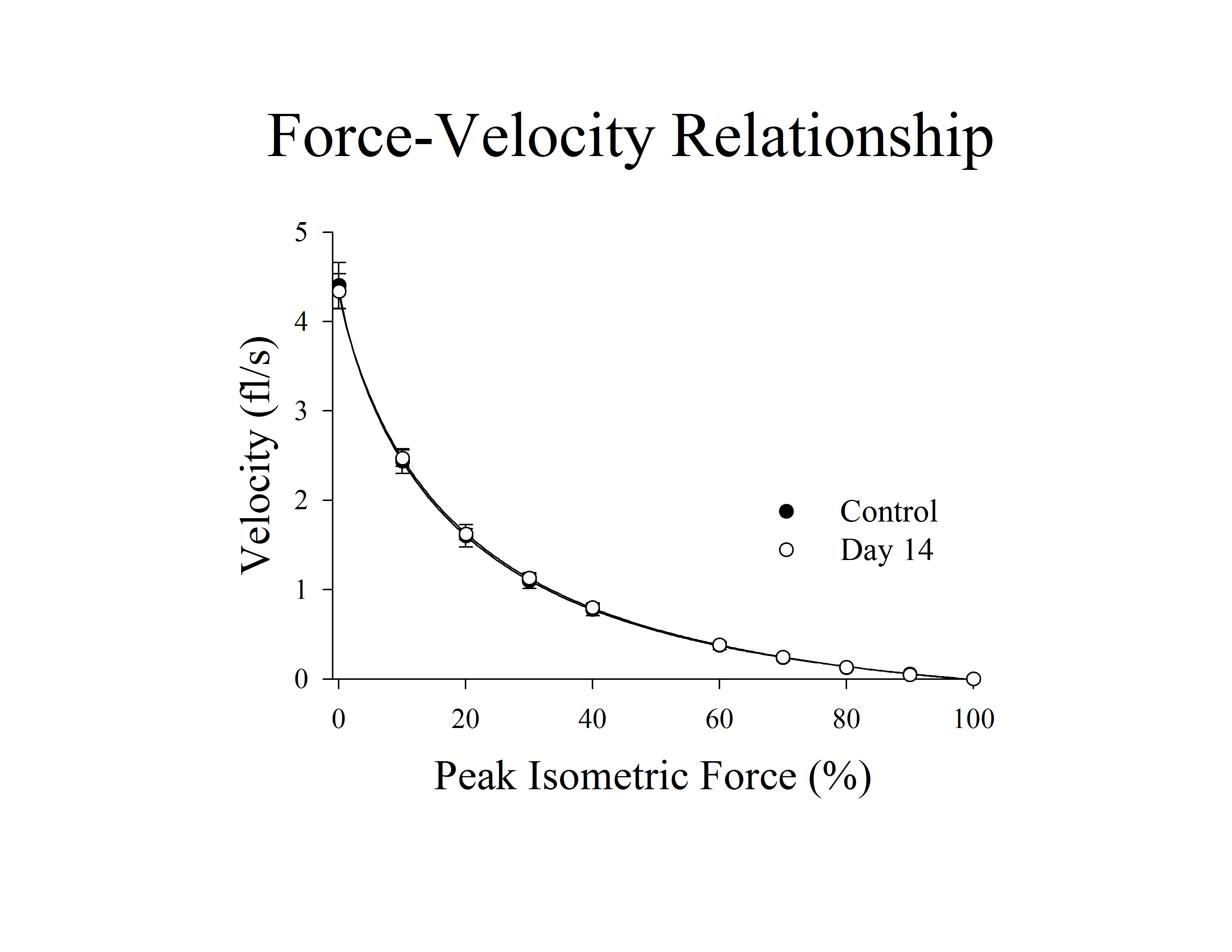

Supplement: S1 Fig — Control was innervated muscle and 14 day was denervated for 14 days. Sample size per group, n = 6–8. Values are mean±SEM. (TIF) [file pone.0160839.s001.tif]
